# Neural tube defects among new borns: a cross-sectional study

**DOI:** 10.3389/fped.2023.1191556

**Published:** 2023-05-22

**Authors:** Mahder Kidane, Yohanes Sime, Anteneh Gashaw, Getachew Chane

**Affiliations:** ^1^ Departments of Medicine, Mizan Tepi University, Mizan, Ethiopia; ^2^ Departments of Psychatry, Dilla University, Dilla, Ethiopia; ^3^ Departments of Midwifery, Dilla University, Dilla, Ethiopia; ^4^ Departments of Anatomy, Jimma University, Jimma, Ethiopia

**Keywords:** prevalence, neural tube defect, newborn, jimma, Ethiopia

## Abstract

**Background:**

Neural tube defects are a major public health issue that contributes significantly to morbidity and mortality, particularly in low-income countries such as Ethiopia. In Ethiopia, particularly in the study setting, there is a paucity of data on the prevalence, magnitude, and associated factors of neural tube defects. As a result, the purpose of this study was to evaluate neural tube defects and associated factors in JUMC.

**Methods:**

This study was an institution-based cross-sectional study conducted from June to September 2021. Data was collected using a structured questionnaire adapted from previous literature. Data were analyzed using SPSS version 26 software. Logistic regression analysis was performed to assess the association between dependent and independent variables. Independent variables with a *p*-value < 0.05 were taken as statistically significant with neural tube defects.

**Results:**

The prevalence of NTDs in this study was 3.6%. Preterm newborns with GA < 34 AOR 2.9(1.2–9.7), newborns with birth weight b/n 1,000–1,499 AOR 5.2(1.1–9.4), born with weight b/n 1,500–2,499 AOR 2.1(1.3–8.7), exposure to smoke AOR 2.1(1.2–8.8), radiation AOR 6.8(1.3–14.5), at least one history of abortion AOR 10.1(7.2–21.0) and mothers with AED intake AOR 5.7(2.3–18.4) were found to be associated significantly.

**Conclusion:**

The results indicated a significant frequency of neural tube abnormalities in newborns. The use of AEDs, abortion, and radiation have all been linked to those NTD cases. Pregnant women are advised to learn more about the need of beginning prenatal care as soon as possible because the aforementioned issues will be addressed during this treatment.

## Background

A neural tube defect (NTD) happens when the neural tube fails to close properly, exposing the neurodevelopment or spinal cord to amniotic fluid (1). Spina bifida, anencephaly (absence of brain calvariates, totally or partially), encephalocele (brain shrink and Calvary defect meninges), craniorachischisis (anencephaly associated with permanent spinal and neural tissue exposure), and anencephaly (dysraphism of the occipital region accompanied by retroflexion of the neck and trunk) are included in the spectrum (2).

NTDs are divided into two types: “open” NTDs where Open” NTDs include craniorachischisis, which results from total neurulation failure and leaves most of the brain and spinal cord open, anencephaly, which occurs when a defect occurs in the cranial region, and spina bifida cystica in the lumbosacral region (3).

Neural tube defects are among the top five most severe birth defects worldwide, and they are associated with significant death, morbidity, and disability. Each year, an estimated 300,000 babies are born with NTDs, resulting in approximately 88,000 deaths and 8,6 million disability-adjusted life years (DALYs) (4, 5). NTDs can account for up to 29% of neonatal deaths due to observed birth defects in low-income countries. In Africa, NTD are the most common birth defects. Around 1–3/1,000 births are affected per year. In Ethiopia, 63.3 cases per 6,910,000 children had an estimated pooled prevalence of neural tube defects (6–8).

Multiple medical and socioeconomic impacts are associated with neural tube defects. They could 71 lead to fetal malformation or stillbirth, and about 50 percent optional termination of pregnancies. Around 75% of living births with an NTD resulted in the deaths of children under 5 and disabilities globally (9). In resource-limited countries where preventive measures and long-term 75 care for surviving patients are restricted, the consequences of NTDs are especially obvious (10).

Life-long physical problems from NTD require lifetime medical attention which adds a considerable burden for the patients concerned, their families, national health services, and governments (11). Parents face high distress when their child is diagnosed with defects during pregnancy, and they must choose between the grief of a termination/stillbirth or the financial and emotional challenges of caring for a child with disabilities (12).

Lifetime direct medical and indirect costs are significant for the affected patients, parents, and families. Prevention ensures that such a multi-factorial burden does not occur. In addition to its burden, stigma towards NTDs by the community has been documented elsewhere in Africa, affecting the quality of life of families through social, economic, and emotional distress (13).

Studies conducted across the globe have identified different risk factors associated with NTDs. These include low socioeconomic status, maternal exposure to certain environmental factors (i.e., chemicals and pesticides), tobacco use during pregnancy, genetic factors, pregnancy in the late maternal age, poor intake of folic acid prior to or during pregnancy, sex of the neonate, and lack of antenatal care (14–17). Furthermore, there exists little evidence from Ethiopia, particularly the study area. As a result, the current study was designed to estimate the level of NTD among newborns.

## Methods and materials

### Study design and period

An institutional-based cross-sectional study design was employed from June 2021 to September 2021, at Jimma university medical center.

### Study setting

The study was conducted at Jimma University Medical Center (JUMC). Jimma University medical center (JUMC) is found in Jimma town, Oromia regional state, which is 352 km from Addis Ababa to the southwest, the capital city of Ethiopia. JUMC is one of the oldest governmental hospitals, which was established in 1937 during the Italian occupation for the service of their soldiers. After the withdrawal of the colonial occupiers, it has been running as a public hospital under the Ministry of Health by different names at different times and is currently named “Jimma University Medical Center”.

### Population

The inclusion criteria of the study were: all newborns and their mothers discovered during data collection. Those newborns with mothers who were seriously ill or unable to communicate during the data collection period were excluded from the study.

### Sample size determination and sampling technique

The sample size was calculated using the single population proportion formula with a 95% confidence interval, a 3% margin of error (because the estimated proportion is small, 20%), the previous study's population proportion of 5.71%, and a 10% non-response rate. The study's final sample size was 253. During the data collection period, study participants were chosen using systematic random sampling.

### Study variables

Neural Tube Defects (presence/absence) was the outcome variable and independent variables include Socio demographic factors mother and new born **(**Maternal Age, Residence, Marital status, Gestational age in weeks, Sex of new born, Birth weight in grams), Perinatal and Behavioral Characteristics of Mothers **(**Place of antenatal care, Mode of delivery, Passive smoking, Radiation exposure, History of abortion or/and still birth, Amount of coffee per day, AEDs intake during pregnancy, Parity, Spouse relation), and **P**hysical Examination of Newborns **(**Consciousness, Tone upper left and right, Tone left and right lower, Anal tone, Moro reflex, grasp reflex, suckling reflex, Sensation right and left lower, Sensation right and left upper, Power right and left lower, Deep tendon reflex right and left lower, Cradle maneuver).

### Data collection method and instrument

Data was collected using a structured questionnaire adapted from previous literature. The questionnaire was first prepared in English and then translated into local languages (Amharic and Afan Oromo) and back into English for consistency by different language experts. Data was then collected by two neonatal nurses and one General Practitioner using face-to-face interviews with maternally related variables (sociodemographic characteristics, labor and delivery-related factors, obstetric and maternal lifestyle, and clinical symptoms of newborns) and physical examination of the newborn by a GP and a thorough review of medical records to obtain birth weight and condition of the newborn before admission.

### Data processing and analysis

The collected data was checked for its completeness and cleaned before entry into the computer. Then, the data was coded, cleaned, edited, and entered into Epi Data version 4.6 and exported to SPSS window version 26 for analysis. Descriptive statistics were presented in frequency, tables, texts, and summary measures. Bivariate and multivariate analyses were done to observe the association between each independent variable and outcome variable by using binary logistic regression. The goodness of fit was checked by the Hosmer-Lemeshow statistic at a *P*-value of greater than 0.05. All variables with *P *< 0.25 in the bivariate analysis were included in the final model of multivariate analysis in order to control all possible confounders. The statistical association was measured by odds ratio with 95% CI. Adjusted odds ratio along with 95% CI was estimated to identify the associated factors with Neural tube defects by using multivariate analysis in binary logistic regression. In this study, a *P*-value < 0.05 was considered statistically significant.

## Result

### Maternal sociodemographic characteristics

From the total of 253 mother neonate pairs requested, all participated in the study giving a response rate of 100%. The mean age of the mothers was 23 ± 5year with rural residents accounting for 67.99%. Of the mothers who participated in the study, 186 (73.51%) were married with the plurality (104, 41.1%) attending up to primary school and 146 (57.7%) having a low monthly income (<2143brr). The majority of newborns were female, accounting for 139 (54.5%). Regardless of sex, 21 (8.3%) were born <34 weeks, 59 (23.3%) b/n 34–36 wks, and the majority were born between 37 and 42 weeks of gestation respectively. Most newborns (174, 68.82%) weighed b/n 2,500–4,000 grams (Table 1).

**Table 1:** Sociodemographic characteristics of mother-newborn pairs at labor ward JUMC, southwestern Ethiopia, in August 2021.

Variable	Category	Frequency(*n*)	Percentage (%)
Age of mother	<18	23	9.09
	18–35	181	71.54
	>35	49	19.37
Residence	Urban	81	32.01
	Rural	172	67.99
Marital Status	Single	48	18.9
	Married	186	73.51
	Widowed	0	0
	Divorced/Separated	18	7.59
Current educational status of mother	Illiterate	83	32.8
	Primary school (1–8)	104	41.1
	Secondary school (9–12)	58	22.9
	Above secondary (TVET, degree…)	8	3.2
Gestational age in weeks	Less than 34	21	8.3
	34–36	59	23.3
	37–42	173	68.4
Sex of newborn	Male	114	45.05
	Female	139	54.5
Birth weight in grams	1,000–1,499	25	9.88
	1,500–2,499	54	21.3
	2,500–4,000	174	68.82

### Perinatal and behavioral characteristics

Regarding perinatal and behavioral characteristics, 89.64% had ANC visits and a majority 163 (64.4%) had follow-up at the health center level. Of all the mothers that participated in the study, 10 (3.95%) of them took folate supplements at or before the first trimester of pregnancy. Only 62 (24.49%) of them were exposed to passive smoking and 23 (9.09%) were exposed to radiation during pregnancy. The majority 212 (83.79%) of the mothers had coffee intake, with 169 (66.7%) of drinkinh less than three cups per day. It was found that 13 (5.12%) of the mothers had a history of abortion and/or stillbirth. Most (150, 59.7%) of the mothers are Multiparous and 3 (1.2%) of them were in a consanguineous relationship (Table 2).

**Table 2:** Perinatal and behavioral characteristics of mothers at labor ward JUMC southwest Ethiopia, August 2021 EC.

Variable	Category	Frequency (*n*)	Percentage (%)
Place of antenatal care	Hospital	54	21.34
	Health center	163	64.4
	Private clinic	10	3.9
	None	36	10.36
Maternal nutrition at the 1st trimester and/or before	Folate supplement(tab)	10	3.95
	Multivitamin	8	3.16
	None	235	92.88
Smoking/passive smoking	Yes	62	24.49
	No	191	75.51
Radiation exposure	Yes	23	9.09
	No	230	90.91
Coffee intake	Yes	212	83.79
	No	41	16.21
History of abortion or/and stillbirth	Yes	13	5.13
	No	240	94.87
Amount of coffee per day	Less than 3 cups	169	66.7
	Three cups or more	43	33.3
Anti-epileptic drug intake during pregnancy	Yes	21	8.3
	No	232	91.7
Parity	Primiparous	92	36.3
	Multiparous	150	59.2
	Grand Multiparous	11	4.5
Spouse relation	Consanguineous	3	1.2
	Non-biologic	250	98.8

### Prevalence of neural tube defect

**Figure 1:**
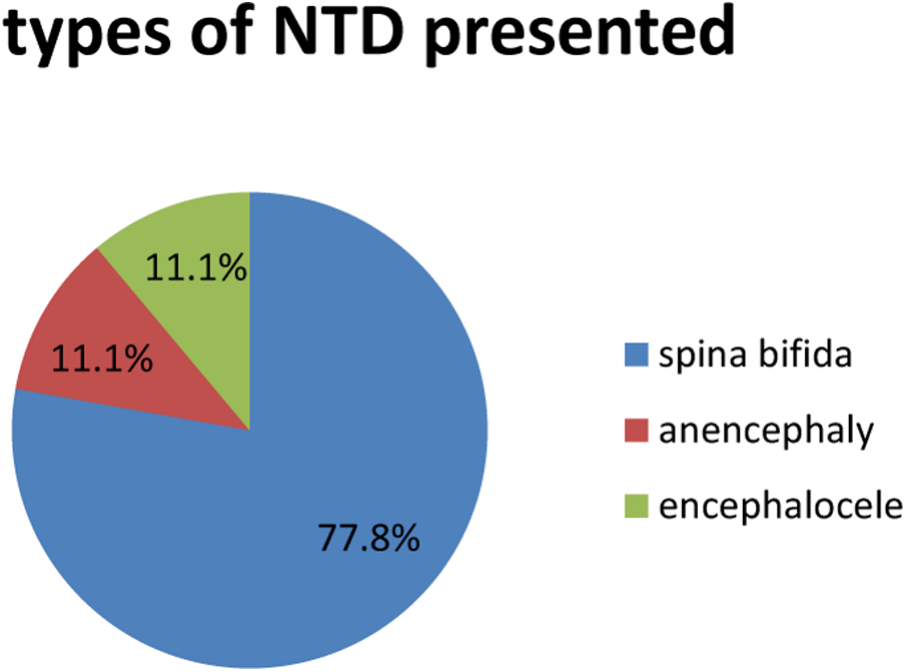
Types of NTDs presented among newborns at Jimma medical center, southwest Ethiopia, 2021.

From 253 newborns, only 9 (3.6%) of them were found to have neural tube defects, with the most common (7, 77.7%) attributed to spina bifida followed by anencephaly and encephalocele, each accounting for 1 (11.1%) NTD. The most common area for spina bifida to be located was the thoracolumbar region (4, 57.4%) (Figure 1).

### Physical examination of newborns

A physical examination done on the affected neonates reveals that consciousness was altered in 2 (22.3%) of them, with an upper limb being hypotonic in 1 (11.2%) and a lower limb being hypotonic in 4 (44.5%). Loose anal tone only affected in 4 (44.5%) of them, with abnormal reflexes, Moro, grasp, and suckling accounting for 2 (22.3%) each (Table 3).

**Table 3:** Physical examination of newborns at labor ward JUMC Southwest Ethiopia August, 2021GC.

Variables	Category	Frequency	Percent
Consciousness	Alert	7	77.7
	Lethargic	2	22.3
Tone upper left and right	Normotonic	8	88.8
	Hypotonic	1	11.2
Tone lower left and right	Normotonic	5	55.5
	Hypotonic	4	44.5
Anal tone	Normotonic	5	55.5
	Loose	4	44.5
Moro reflex	Complete	7	77.7
	Incomplete	2	22.3
Grasp reflex	Strong	7	77.7
	Weak	2	22.3
Suckling reflex	Sustained	7	77.7
	Un-sustained	1	11.1
	Absent	1	11.1
Sensation right and left lower	Intact	8	88.8
	Lost	1	11.2
Power right and left lower	Less than 3/5	2	22.3
	More than 3/5	7	77.7
Cradle maneuver	Positive	6	66.6
	Negative	3	33.4

### Factors associated with neural tube defects

Bivariate regression analysis was done to see factors associated with neural tube defects. Hence, GA in weeks, Birth weight, maternal nutrition, exposure to smoke, radiation, Amount of coffee intake per day, Residence, history of abortion, and AED intake during pregnancy were found to be associated with neural tube defect and were inserted into multivariate regression analysis; meanwhile, in multivariate regression analysis GA, birth weight, exposure to smoke, radiation, history of abortion and AED intake during pregnancy were found to be significantly associated with neural tube defects. Preterm newborns with GA < 34 have a 3 times greater chance of having an NTD with an AOR of 2.9 (1.2–9.7).

Newborns with birth weight b/n 1,000–1,499 have approximately five times more chance with AOR 5.2 (1.1–9.4) of having neural tube defect while those born with weight n/n 1,500–2,499 have 2 times the chance of NTD with an AOR of 2.1 (1.3–8.7). Exposure to smoke and radiation were found to have the likelihood of association with developing NTD two times with AOR 2.1(1.2–8.8) and 7 times with AOR 6.8 (1.3–14.5) respectively. Furthermore, mothers with a history of at least one abortion had ten times the chance of having a baby with NTD, with AOR 10.1(7.2–21.0), and mothers with AED intake have approximately 6 times more chance of having a baby with NTD, with AOR 5.7(2.3–18.4) than their counterparts (Table 4).

**Table 4:** Bivariate and multivariable factors associated with NTDs at labor ward JUMC in August, 2021 GC.

Variables	Categories	Neural tube defect		COR (95%CI)	AOR (95%CI)	*p*-value
		Yes N%	No N%			
GA in weeks	<34wk	2 (9.5)	19 (90.5)	3.82 (1.2–10.4)	2.9 (1.2–9.7)	.001^**^
	34–36	3 (5.1)_	56 (94.9)	0.39 (0.13–1.2)	0.12 (0.04–0.5)	.30
	37–42wk	4 (2.3)	169 (97.7)	1	1	
BW in gram	1,000–1,499	1 (4.0)	24 (96.0)	5.2 (1.1–9.4)	4.6 (1.6–10.7)	.015^*^
	1,500–2,499	2 (3.7)	52 (96.3)	2.6 (1.1–7.2)	2.1 (1.3–8.7)	.001^**^
	2,500–4,000	6 (3.4)	168 (96.6)	1	1	
Maternal nutrition	Folate	2 (2.0)	8 (98)	6.00 (2.1–12.6)	4.5 (2.3–12.7)	.304
	Multivitamin	2 (33.3)	6 (66.7)	5.6 (1.6–13.2)	3.2 (0.9–13.9)	.28
	None	5 (2.1)	230 (97.9)	1`	1	
Passive exposure to cigarette	Yes	3 (4.8)	59 (95.2)	3.2 (1.1–7.3)	2.1 (1.2–8.8)	.015^*^
	NO	6 (3.1)	185 (96.9)	1	1	
Radiation exposure	Yes	4 (17.3)	19 (82.7)	8.9 (1.2–12.3)	6.8 (1.3–14.5)	.001^**^
	NO	5 (2.1)	225 (97.9)	1	1	
Amount of coffee per day	<3 times	3 (1.8)	166 (98.2)	1	1	.649
	>3 times	6 (13.9)	37 (86.1)	1.7 (0.4–5.2)	0.48 (0.3–3.8)	
Residence	Urban	2 (2.5)	79 (97.5)	1	1	.688
	Rural	7 (4)	165 (96)	0.89 (0.67–1.19)	1.06 (0.79–1.41)	
Hx of abortion	Yes	5 (38.4)	86 (1.6)	12.3 (6.8–18.9)	10.1 (7.2–21.0)	.015^*^
	No	4 (1.7)	236 (98.3)	1	1	
AED intake during pregnancy	Yes	4 (19.0)	17 (81.0)	9.7 (4.3–18.2)	5.7 (2.3–18.4)	.002^*^
	No	5 (2.2)	227 (97.8)	1	1	

COR, crude odds ratio; AOR, adjusted odds ratio; CI, confidence interval.

* Significant at *P* < .05.

** Significant at *P* < .001.

## Discussion

This study assessed the prevalence of neural tube defects and their associated factors among newborns in Jimma medical center. The overall magnitude of NTD in this study was found to be 3.6% (95%CI: 2.29–5.65) among newborns at Labor Ward JUMC in southwest Ethiopia. Spina bifida accounted for the majority (77.8%) of cases, and anencephaly and encephalocele accounted for 11.1% each. This is comparable to the study done at NICU in HFSUH Eastern Ethiopia having a prevalence of 5.71%; of these, 20 (83.5%) and 4 (16.5%) had spina bifida and encephalocele respectively (18). NTDs were defined as cases of anencephaly, spina bifida, and encephalocele based on ICD-10 criteria. During seven months, they observed 55 cases of NTDs out of 8,677 births after 28 weeks of gestation—birth prevalence of 63.4 per 10,000 births [95% confidence interval (CI), 51–77] (14).

Based on our study and prior studies as noted above, the rate of NTDs is much higher than that reported in Latin America where the prevalence of neural tube defects was 4.73 per 1,000 deliveries (89:18,807). The most common neural tube defects were myelomeningocele (47.2%), anencephaly (26.9%), and encephalocele (16.9%) (19). Additionally, our study finding is higher than the study done on Neural tube defects among neonates delivered in Al-Ramadi Maternity and Children's Hospital, western Iraq, where 33 infants were delivered with NTDs, giving an incidence of 3.3/1,000 births. The most prevelant NTD types were myelomeningocele and anencephaly at thoracolumbar and lumbosacral sites (20).

The possible explanation for the difference might be differences in socio-cultural and environmental factors, availability of health services, and low attention given to NTDs in developing countries including ourown. In addition to this low sample size in this study might be a possible explanation for the difference observed.

Factors associated with NTDs were identified by multivariate logistic regression analysis. According to the study analysis GA, birth weight, exposure to smoke, radiation, history of abortion/stillbirth, and AED intake during pregnancy were factors associated with NTD.

The study reveals that Preterm newborns with GA < 34 have 3 times the chance of having an NTD. Newborns with birth weight b/n 1,000–1,499 have approximately a five times greater chance of having a neural tube defect while those born with weight b/n 1,500–2,499 have a 2 times higher chance of NTD. These factors are supported or consistent with the studies done in Hiwot Fana (18), Ethiopia, and three teaching hospitals in Addis Ababa (14).

Exposure to smoke and radiation were found to have a likelihood of association with developing NTD two and seven times greater than the baseline, respectively. This is supported by studies carried out in China (21) and Turkey (22).

Mothers with AED intake have approximately 6 times more chance of having a baby with NTD than their counterparts. The studies conducted in the Netherlands (23) and western Ethiopia (24) also support this finding.

Furthermore, mothers with history of at least one abortion had ten times the chance of having a baby with NTD. This finding is supported by studies in China (21), Saudi Arabia (25), and Iran (26).

## Conclusion

The burden of neural tube defects was 3.6 percent among newborns in Jimma medical center. It is preventable if mandatory fortification of foods with folic acid is initiated before conception. NTDs have been associated with gestational age and birth weight, AED intakes during pregnancy, abortion history or mortality, and exposure to radiation. Our findings recommend that pregnant mothers become more aware of the timely introduction of prenatal care because the above factors will be tackled in the course of this care. More attention is needed to monitor NTDs in southwest Ethiopia. This study highlights the urgent need for further interventional studies to develop innovative solutions to improve outcomes in the study settings.

## Data Availability

The raw data supporting the conclusions of this article will be made available by the authors, without undue reservation.
